# Relationship between the Molecular Coil Dimension and the Energy Storage Modulus of Polymer Solution Configured with Oilfield-Produced Sewage

**DOI:** 10.1155/2020/2538521

**Published:** 2020-06-22

**Authors:** Peng Wang, Wenguo Ma, Yunbao Zhang, Qiuyan Yan

**Affiliations:** ^1^Guangzhou Institute of Geochemistry, Chinese Academy of Science, Guangzhou 510640, China; ^2^University of Chinese Academy of Science, Beijing 100049, China; ^3^Sixth Oil Production Factory, Daqing Oil Field, PetroChina, Daqing 163318, China; ^4^Key Laboratory of Enhancing Oil and Gas Recovery of Education Ministry, School of Petroleum Engineering, Northeast Petroleum University, Daqing 163318, China; ^5^Bohai Oil Research Institute, Tianjin Branch of CNOOC Ltd., Tianjin 300450, China; ^6^Institute of Chemical Engineering, Northeast Petroleum University, Daqing 163318, China

## Abstract

Polymer viscoelastic solution is the non-Newtonian fluid and widely used in oil production. In the process of seepage, the mechanism of the polymer solution with different molecular coil dimensions (Dh) flooding on remaining oil is unknown. By using the dynamic light scattering instrument, the molecular coil dimension of the polymer solution is tested. By using the HAAKE rheometer, the creep recovery test data of the polymer solution under the same creep time condition are obtained. The effects of polymer solutions with different Dh on residual oil are observed, by using the visible pore model. The results show that the higher the molecular weight (*M*_w_) of the polymer, the larger the size of the molecular coil dimension. The elasticity characteristics of the polymer solution are sensitive to the molecular coil dimension. As Dh of polymer molecules becomes larger, the contribution of the elastic portion to the viscosity of the polymer solution increases. The higher the *M*_w_ of polymer is, the longer the molecular chain is and the size of Dh is larger. On the condition of the polymer solution with different *M*_w_ with 2.5 g/L, when Dh is between 320.0 nm and 327.8 nm, the ratio of the elastic part in the apparent viscosity exceeds the proportion of the viscous part, and the polymer solution composition after these data can be used as a comparative study of elasticity for residual oil use. In the visible pore model, the pore-throat ratio is 3.5, the *E*_R_ of water flooding is 54.26%, the *E*_R_ of the polymer solution with Dh = 159.7 nm is 75.28%, and the increase of *E*_R_ is 21.02% than that of water flooding. With the increase of Dh to 327.8 nm, the final *E*_R_ of the experimental polymer solution is 97.82%, and the increase of *E*_R_ of the polymer solution than that of water flooding is 43.56%. However, in the model with a pore-throat ratio of 7.0 and the same polymer solution with Dh = 327.8 nm, the increase of *E*_R_ of the polymer solution is only 10.44% higher than that of water flooding. The effect of the polymer solution with the same Dh is deteriorated with the increase of the pore-throat ratio.

## 1. Introduction

With the gradual expansion of the application scale of polymer flooding and composite system, the water resources used for the preparation of the polymer solution are becoming increasingly tense; at the same time, a large number of produced water bring great pressure on environmental protection [1–4]. The viscoelastic properties of the polymer solution are the key factors affecting oil recovery and the type of residual oil [5–9]; the test results of the geometrical form of the polymer molecules in the polymer system configured with the treated sewage directly affect the polymer system and the pore adaptability, thereby affecting the microscopic residual oil use effect [10–14]. In this paper, the use of oilfield-produced sewage to prepare the polymer solution has become an inevitable choice for the oilfield production. By using the dynamic light scattering instrument, the viscosity, elasticity, and the molecular coil dimension (Dh) are studied. As an effective parameter, the creep recovery test can be used to study the viscoelasticity of the polymer solution. The relationship between molecular storage modulus of the polymers (prepared by the oilfield-produced water) is analyzed, and both the *M*_w_ and the concentration of polymer (*C*_p_), combined with the core model designed by the pore structure of the actual reservoir, guide the hydrolysis of the indoor oil flooding experiment, and the changes of micro remaining oil in sandstone of pore size level is studied, and the best polymer system for oil displacement suitable for experimental pore construction parameter data is given.

## 2. Test Instruments for Molecular Coil Dimension and Creep Recovery

### 2.1. Test Instruments

**Figure 1:**
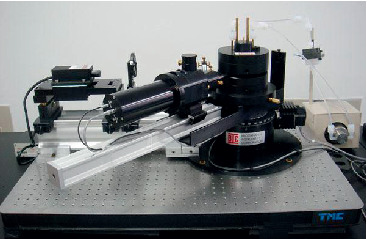
BI-200SM wide angle dynamic/static light scattering system.

The instrument used is a wide angle dynamic/static light scattering system type BI-200SM (Brookhaven Instruments Corp, USA),as shown in Figure 1; the main components of the system include a laser correlator (Type BI-9000AT), signal processing apparatus, and argon ion laser (power: 200 mW and wave length: 532.0 nm). The dynamic light scattering instrument used in this research can directly test Dh. Dh can be used to characterize the crimping degree of polymer chains and molecular coil dimension.

**Figure 2:**
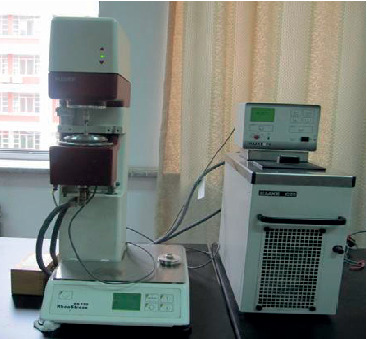
HAAKE RS150 rheometer.

The instrument HAAKE RS150 rheometer (Germany) used in this test for creep recovery is shown in Figure 2. The constant temperature is the water temperature s at 45°C in this test, and data are automatically controlled by a computer. The C60/1Ti cone plate is adopted in the test (Taper: 1°), the shear rate itables 0.1∼1000 (1/s), and the cone gap of the test system is 0.052 mm.

### 2.2. Creep Recovery Test Principle

**Figure 3:**
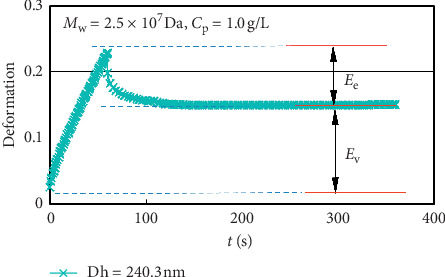
Creep recovery curves of the polymer solution with different Dh (0.5 g/L).

In the creep recovery experiment, as shown in Figure 3, during the creep period (time from 0 s to 60 s), with a constant stress (stress = 0.005 Pa) applied, the straining increases with time. The stress is removed after 60 s; until 360 s is the recovery period, the straining decreases sharply first and then reach a stable value. The stable straining (*γ*_ss_) is the contribution of the viscosity parts of the polymer solution, and the recoverable straining (*γ*_max_ − *γ*_ss_) is the contribution of the elasticity parts. The percentage of total straining (*γ*_max_) can be decomposed into viscosity parts and elasticity parts. The proportion of the straining contributed by elasticity parts (*E*_e_) and the strain contributed by viscosity parts (*E*_v_) to the total straining reflect the elasticity and viscosity of the viscoelastic polymer solution; the calculation formula is shown in the following formula:

(1) $$\begin{array}{c} E_{\text{v}}=\frac{\gamma_{\text{ss}}}{\gamma_{\max}}\times 100\%,\\ E_{\text{e}}=\frac{\gamma_{\max}-\gamma_{\text{ss}}\;}{\gamma_{\max}}\times 100\%. \end{array}$$

### 2.3. Test Results and Analysis

**Figure 4:**
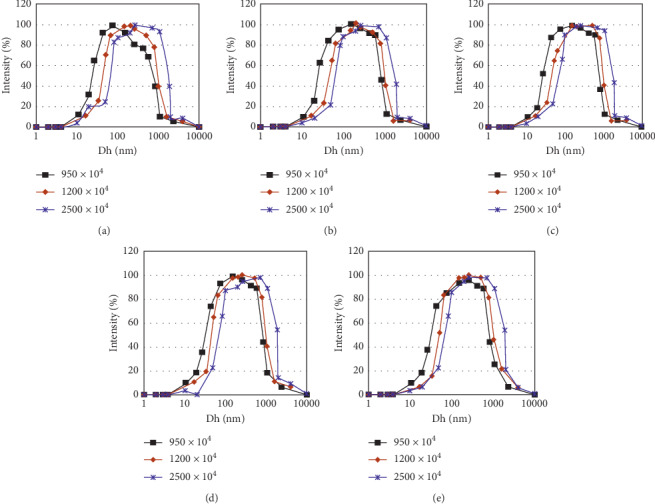
Distribution curve of Dh with different *M*w and polymer concentrations. (a) Concentration, 0.5 g/L. (b) Concentration, 1.0 g/L. (c) Concentration, 1.5 g/L. (d) Concentration, 2.0 g/L. (e) Concentration, 2.5 g/L.

Figure 4 shows the Dh distribution of different *M*_w_ with a polymer concentration of 0.5, 1.0, 1.5, 2.0, and 2.5 g/L.  Table 1 shows the Dh equivalent peak of different *M*_w_ with the polymer of different concentrations.

**Figure 5:**
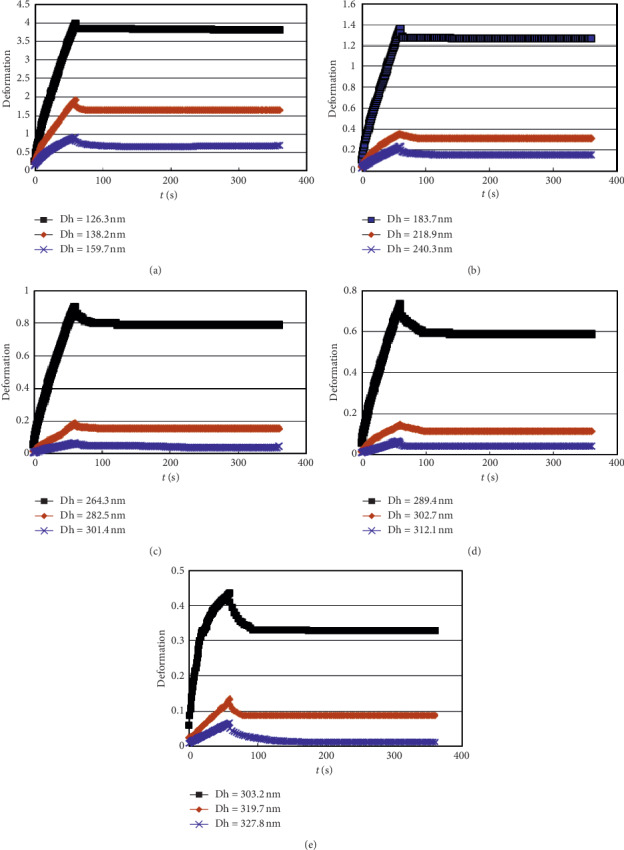
Creep recovery curves of the polymer solution with different Dh. (a) 0.5 g/L. (b) 1.0 g/L. (c) 1.5 g/L. (d) 2.0 g/L. (e) 2.5 g/L.

Figure 5 shows the result of the creep recovery test of the polymer solution under the same creep time condition, which includes the *M*_w_ of 950 × 10^4^, 1200 × 10^4^, and 2500 × 10^4^ with the *C*_p_ from 0.5 to 2.5 g/L.

**Figure 6:**
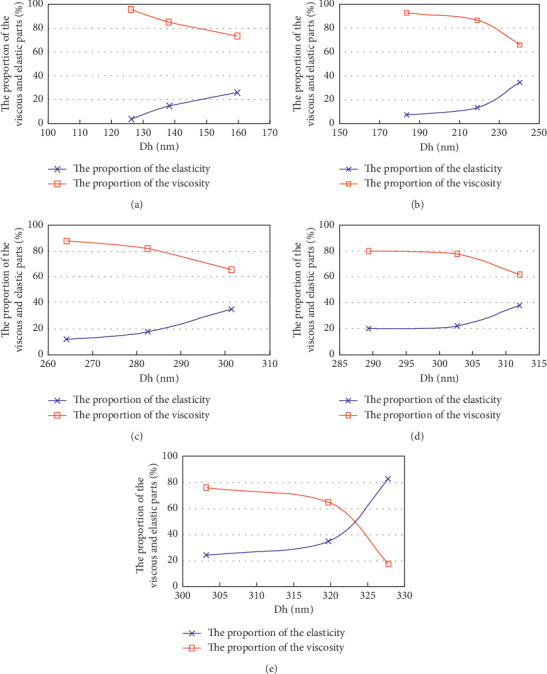
The proportion of the viscosity and elasticity parts of different Dh. (a) 0.5 g/L. (b) 1.0 g/L. (c) 1.5 g/L. (d) 2.0 g/L. (e) 2.5 g/L.

Figure 6 and Table 1 show the proportion of the viscosity and elasticity parts with different Dh, *M*_w_, and *C*_p_. It can be seen from the creep recovery test results that with the increase of the molecular coil dimension, deformation decreases gradually and the proportion of the elastic part of the solution increases gradually and even exceeds the viscous part. This is due to the increase of the polymer molecular coil dimension. The molecules in the system are more easy to wind together and the ability to resist the external force is strong, at the same time due to the increase of electrostatic repulsion within the molecular coil; so, it is not easy to be deformed.

From the Dh distribution curve of different *M*_w_ with same *C*_p_, we can see that, the higher the *M*_w_ of polymer is, the longer the molecular chain and the more complex the conformation is, and the size of Dh is larger. On the condition of the polymer solution with different *M*_w_ with 2.5 g/L, when Dh is between 320.0 nm and 327.8 nm, the ratio of the elastic part in the apparent viscosity exceeds the proportion of the viscous part, and the polymer solution composition after these data can be used as a comparative study of elasticity for residual oil use.

## 3. Oil Displacement Experiments and Analysis

The effects of different elastic polymer solutions with different Dh on residual oil are observed, using the visible pore model.

### 3.1. Experimental Materials and Experimental Conditions

The experimental temperature is 45°C, and the experimental oil is a type of simulated oil prepared from crude oil (viscosity value is 10.0 MPa·s, 45°C).

The *M*_w_ of polyacrylamide (HPAM) used in the experiments is 2500 × 10^4^, and the concentration of the polymer solution is spate 0.5 g/L, 1.5 g/L, and 2.5 g/L.

Salinity mineralization of water used in experiments is 6.778 g/L.

The model used in the visual experiment is a transparent glass homogeneous core etched according to the actual core pore structure. The model have two kinds: model 1 has a pore size of 105.0 microns and a throat size of 30.0 microns; the pore throat ratio of model 1 is 3.5. Model 2 has a pore size of 210.0 microns and a throat size of 30.0 microns; the pore throat ratio of model 2 is 7.0. The injection rate is 0.02 ml/hr.

### 3.2. Experimental Procedure

1. The microscopic model injects oil
2. The displacement speed of the simulated oil layer is constant speed water flooding, and displace the water until the oil is not seen at the exit
3. Inject the viscoelastic polymer solution at a constant speed to drive the oil, record the dynamic image during the displacement process, and record the images before and after the flooding
4. Calculate the oil displacement efficiency (*E*_R_) under each displacement condition
5. Clean the core and finish the experimental equipment
6. Continue the experiment changing the concentration of the polymer solution and repeat the first five steps

### 3.3. Experimental Results and Analysis

#### 3.3.1. Rheological Parameters of the Polymer Solution

**Figure 7:**
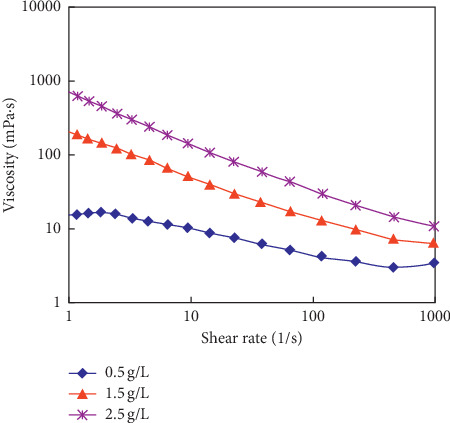
The curve of the polymer solution with different *C*_p_ (*M*_w_ = 2500 × 10^4^).

**Figure 8:**
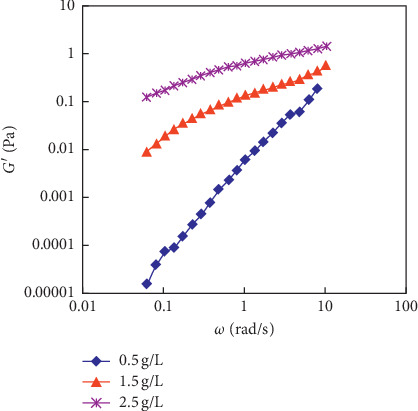
The storage modulus of the polymer solution with different *C*_p_ (*M*_w_ = 2500 × 10^4^).

**Figure 9:**
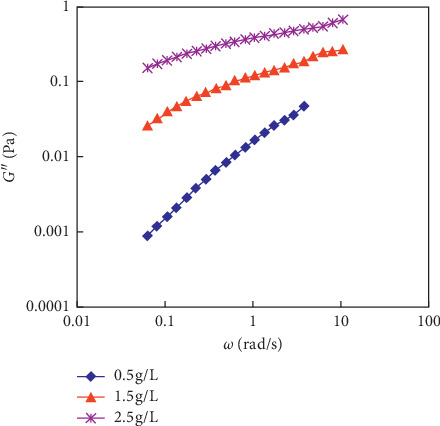
The energy consumption modulus of the polymer solution with different *C*_p_ (*M*_w_ = 2500 × 10^4^).

The rheological curves of the polymer solution system are shown in Figure 7, the determined *M*_w_ of the polyacrylamide (HPAM) used in the experiments is 2500 × 10^4^, and the concentrations of polymer solution (C_p_), respectively, are 0.5 g/L, 1.5 g/L, and 2.5 g/L. The storage modulus (G′) and dissipation modulus (G″) of polymer solutions are tested. The test results are shown in Figures 8 and 9. It can be ground from the viscous relationship curve, and the viscosity gradually increases by increasing the concentration of the polymer.

#### 3.3.2. Analysis of Oil Displacement Experiment Results

**Figure 10:**
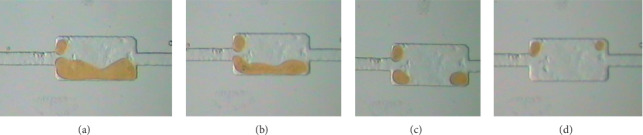
Distribution of residual oil after displacement with different Dh (pore-throat ratio 3.5). (a) Water flooding. (b) Dh = 159.7 nm. (c) Dh = 301.4 nm. (d) Dh = 327.8 nm.

**Figure 11:**
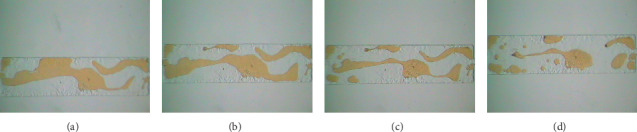
Distribution of residual oil after displacement with different Dh (pore-throat ratio 7.0). (a) Water flooding. (b) Dh = 159.7 nm. (c) Dh = 301.4 nm. (d) Dh = 327.8 nm.

The *E*_R_ results of oil displacement experiments for the polymer solution with different Dh under different pore-throat ratio visual models are shown in Table 2. The results of residual oil in the visual pore model are shown in Figures 10 and 11.

In the visible pore model, the pore-throat ratio is 3.5, the *E*_R_ of water flooding is 54.26%, the E_R_ of the polymer solution with Dh = 159.7 nm is 75.28%, and the increase of *E*_R_ of the polymer solution is 21.02% than that of water flooding. With the increase of Dh of 327.8 nm, the final *E*_R_ of the experimental polymer solution is 97.82%, and the increase of *E*_R_ of the polymer solution than that of water flooding is 43.56%. However, in the model with a pore-throat ratio of 7.0, the same polymer solution with the Dh = 327.8 nm, the increase of *E*_R_ of the polymer solution is only 10.44% higher than that of water flooding. The effect of the polymer solution with the same Dh is deteriorated, as the pore-throat ratio increases.

The experimental results show that, in the same pore-throat ratio model, as the Dh of polymer solution increases, the residual oil in the visual pore model gradually decreases. As the pore-throat ratio increases, the *E*_R_ of polymer solution with the same Dh gradually deteriorates, and a polymer solution with a larger Dh is needed. The residual oil displacement pictures show that under the same flow velocity conditions and the same pore throat conditions, the creep recovery conditions after the polymer macromolecules flow out of the throat are similar. The deformation of the modulus part restores the force acting on the external stress feedback, and the shear force on the Newtonian fluid in the pores gradually increases.

## 4. Conclusions

The following conclusions were made on the basis of the experimental work performed:

1. The higher the *M*_w_ of the polymer, the larger the size of the molecular coil dimension.
2. The elasticity characteristics of polymer solution are sensitive to the molecular coil dimension. As Dh of the polymer molecules becomes larger, the contribution of the elastic portion to the viscosity of the polymer solution increases.
3. In the same pore-throat ratio model, as the Dh of polymer solution increases, the residual oil in the visual pore model gradually decreases. As the pore-throat ratio increases, the *E*_R_ of polymer solution with the same Dh gradually deteriorates, and a polymer solution with a larger Dh is needed.
4. Under the same flow velocity conditions and the same pore throat conditions, the deformation of the modulus part restores the force acting on the external stress feedback, and the shear force on the Newtonian fluid in the pores gradually increases.

## Figures and Tables

**Table 1:** *E*_e_ and *E*_v_ with different Dh equivalent peaks.

*M*_w_ (×10^4^)	*C*_P_ (g/L)	Dh (nm)	*E*_e_ (%)	*E*_v_ (%)
950	0.5	126.3	4.12	95.88
	1	183.7	7.36	92.64
	1.5	264.3	12.11	87.89
	2	289.4	20.1	79.9
	2.5	303.2	24.4	75.6

1200	0.5	138.2	14.96	85.04
	1	218.9	13.43	86.57
	1.5	282.5	18	82
	2	302.7	22.42	77.58
	2.5	319.7	35.02	64.98

2500	0.5	159.7	26.23	73.77
	1	240.3	34.21	65.79
	1.5	301.4	34.32	65.68
	2	312.1	38.24	61.76
	2.5	327.8	82.7	17.3

**Table 2:** Oil displacement efficiency of the polymer solution with different Dh (*M*_w_ = 2500 × 10^4^).

Pore-throat ratio	*C*_p_ (g/L)	0.0	0.5	1.5	2.5
	Dh (nm)	—	159.7	301.4	327.8

3.5	*E*_R_ (%)	54.26	75.28	88.15	97.82
	*E*_R_ improvement (%)	—	21.02	33.89	43.56

7.0	*E*_R_ (%)	50.41	52.93	54.31	60.85
	*E*_R_ improvement (%)	—	2.52	3.9	10.44

*E *_R_ improvement = *E*_R_ (polymer flooding) − *E*_R_ (water flooding).

## Data Availability

Both the molecular coil dimension data and creep recovery data of the polymer solution used to support the findings of this study are included within this article. The oil displacement experiment data of the visible pore model used to support the findings of this study are currently restricted by the important national science and technology specific projects in order to protect the privacy of the related patients. However, all these data are available from Wenguo Ma (Email: mawenguo110@163.com) for researchers who meet the criteria for access to confidential data.
